# NHS Consultants and Their Use of Private Medical Insurance

**DOI:** 10.7759/cureus.103466

**Published:** 2026-02-12

**Authors:** Craig Nightingale

**Affiliations:** 1 Plastic and Reconstructive Surgery, University Hospitals Coventry & Warwickshire, Coventry, GBR

**Keywords:** consultant survey, nhs funding, nhs staffing, private healthcare sector, private medical

## Abstract

Introduction: NHS (National Health Service) waiting times and workforce pressures have increased in recent years, alongside growth in the UK private healthcare sector. NHS hospital consultants have detailed knowledge of NHS systems and, in some cases, experience of private practice. However, there is limited evidence regarding the extent to which consultants themselves purchase private medical insurance (PMI), or their reasons for doing so. This study aimed to explore rates of self-funded (opposed to employer-provided) PMI ownership among NHS hospital consultants and to examine reported motivations for purchasing PMI.

Methods: An anonymous, cross-sectional online survey was distributed to NHS hospital consultants in England via Royal Colleges and professional networks. The survey collected information on specialty, geographical region, age group, ownership of self-funded PMI, reasons for purchasing PMI, and engagement in private practice.

Results: A total of 445 responses were received, representing approximately 0.7% of NHS hospital consultants in England. Forty-five respondents (10.1%) reported holding self-funded PMI. The most common age group among consultants with PMI was that of 45-54 year olds (42.2%). Psychiatrists, orthopaedic surgeons, and paediatricians accounted for the highest numbers of respondents with PMI. The most frequently cited reason for purchasing PMI was faster access to healthcare (35.6%), followed by peace of mind, family coverage, and perceived quality of care.

Conclusion: In this exploratory survey, self-funded PMI ownership among responding NHS hospital consultants appeared higher than published estimates for the UK general population. Faster access to healthcare was the most commonly reported motivation. Given the low response rate and potential for selection bias, these findings should be interpreted cautiously and may not be truly representative of the group as a whole. Further studies with larger, more representative samples are required to better understand healthcare access perceptions among senior NHS clinicians.

## Introduction

Consultant doctors are often in a privileged position to understand the intricacies of the NHS (National Health Service), many of whom also have experience in the private sector. They, therefore, could be considered to have an important perspective on and insight into matters of access, opportunity, and provision of healthcare.

Since its establishment in 1948, the NHS has had to evolve with an ever-growing population, higher levels of expected care, and at times, increasing costs of medicines and technology, as well as variable funding. What was once considered one of the "jewels in the British crown" is now regularly coming under scrutiny in the media, with growing concerns that it is being stretched too far. Various factors are commonly cited as being potential failings of the NHS, and some of these are explored within this study.

Waiting times have increased across most services since 2008, and peaked in 2023, with 7.7 million people on the referral to treatment (RTT) waiting list [1]. COVID-19 was a hugely influential factor in this, and must be considered when reviewing these figures. This did, however, reduce to 7.4 million by August 2025 [1]. Furthermore, the number of people waiting over a year was roughly 309,000 in 2023 and had reduced to just over 200,000 in early 2025; this number was negligible prior to COVID-19 [1]. There is significant variation in RTT between specialities. Whilst all specialities had over 80% of their RTTs under the 18-week standard prior to COVID-19, it appears that now none of them are above 76%, with Elderly Medicine scoring the best, and Ear, Nose and Throat, Oral Surgery, and Plastic and Reconstructive Surgery all scoring worst, at around 50% [1]. Waiting times and speed of access to care are often cited as reasons why Britons may consider purchasing their own private medical insurance (PMI) [2].

To try and keep up with demand, the annual cost to run the NHS has increased dramatically from just under £280 million in 1948 [3], which equates to about £8.9 billion today when factoring in inflation [4]. This rose to £182 billion in 2024 [5], which is an increase of 1945%, in real terms, over the 76 years since the start of the NHS. This increase has been significantly greater, year on year, than population growth, with the population of England and Wales in 1948 being roughly 41.5 million people [6], and 61.8 million people in 2024 [7], a 33% increase. Furthermore, as a proportion of GDP, the UK has increased NHS funding from 3.5% of GDP in 1948 [8] to 10.9% in 2022 [9]. There are many factors affecting the NHS budget requirements, including care requirements, cost of medicines and technology, as well as the evolution of how healthcare is delivered and monitored. However, it is clear that the NHS is currently funded at significantly higher rates than it has been in the past.

The population of England and Wales grew by 706,000 (1.2% increase) in the year leading to mid-2024, according to Office for National Statistics (ONS) figures [7]. Over that period of time, the number of doctors in England and Wales has increased by about 6%. In 1948, the NHS employed 11,940 doctors, and 125,752 full-time nurses and midwives. These increased to 146,387 [10] and 444,451 [11], respectively, by 2024. This is the equivalent of one doctor per 3476 people, and one nurse per 330 people in 1948, and one doctor per 422 people, and one nurse per 139 people in 2024. This suggests a far improved ratio of doctors to population over the years; this may be an oversimplified statistic and may be confounded by numerous issues, including an increase in services provided by the NHS, the aging nature of the population who require greater amounts of healthcare, evolving aetiology of illnesses (i.e., decrease in infectious causes of death and morbidity, increase in cardiovascular and cancer causes) [12], advancements in medical technology and drug development, and reduced child mortality rate.

Despite these figures, NHS England has reported a 6.7% job vacancy rate (100,023 vacancies) as of September 2025 [13]. This has been improving in recent years, and was 7.4% the previous year. Furthermore, these changes in staffing and funding have not been linear; increases and decreases have varied throughout the years depending on government and economical factors.

UK NHS hospital beds have also reduced dramatically, from about 480,000 [14] in 1948 to under 103,277 in 2024 [15]. However, this is also confounded by the evolution of healthcare over that period, including reduced length of stay for inpatients, more community-based care, more day case treatment, and technological advances.

Private healthcare may be seen by some as an alternative to NHS care. It can provide most of the services available on the NHS, often within relatively short timeframes. However, it is not without its limits, and the vast majority of the UK population still rely on the NHS for their routine, elective, and emergency healthcare needs.

As of 2025, approximately 7.6 million people (12.3% of the UK population) have their own PMI policy, which has increased from 6.7 million in 2020 (13.4% increase) [16]. Around 80% of all PMI claims are through policies provided through employer schemes, estimating that around 2.5% of the UK people pay for healthcare insurance themselves [17]. Highest rates of cover are amongst 35-44 year olds (18%), and 45-54 year olds (19%) [16]. In 2023, the private healthcare industry was valued at £7.59 billion, which is a 12% year-on-year increase in recent times [18].

There are many reasons, including both personal and practical, why someone might purchase PMI. Peace of mind, comfort, and privacy, and also family security have been recorded as key personal reasons for people taking out these policies. From a practical point of view, the most common reasons were to avoid NHS waiting times, greater freedom of choice of specialist and hospital, access to non-NHS treatments, as well as employer-provided cover and international cover [2].

For individuals taking out their own PMI, costs can vary greatly, depending on factors such as age, lifestyle choices, and pre-existing medical conditions, as well as where one lives and the extent of coverage one requires. For healthy adults in their 30s-40s, this is often in the range of £60-95 per month; however, older individuals in the 60-69 year age range may see their policies costing closer to £130-220 per month [19].

This paper aims to examine the rates of NHS hospital consultants who have bought health insurance and to understand the reasons why they may feel the need for extra cover, beyond what the NHS can offer. To our knowledge, no such survey of UK NHS hospital consultants has previously been done.

## Materials and methods

We created a short six-question survey with the following questions: "Which speciality do you work in?", "Which region of the country do you live in?", "What is your age?", "Do you have private healthcare (not otherwise provided by an employer)?", "What is your main reason for getting private health insurance?", and "Do you work in the private sector yourself?" Various levels of private healthcare insurance are available for purchase. This study did not distinguish between these in the survey. Furthermore, those who work in the private sector may do so in various capacities. The assumption in this study is that these consultants are delivering healthcare beyond the NHS and are paid to do so outside of their NHS contracts.

Multiple-answer options were given for the answer regarding the main reasons people purchased PMI; based upon common reasons that various insurers gave as to why people took out their policies, we also included an option for "Other" to measure if we had captured the majority of valid reasons. It was only possible to choose one option, and respondents were asked for their main reason for purchasing PMI. This is an exploratory survey, and as such is not a validated design. However, common reasons cited by insurance companies are a logical and reasonable starting point.

The British Medical Association (BMA) lists 65 different medical and surgical specialities. To simplify the questionnaire, the available options were not fully comprehensive, and smaller specialities were included in subsections such as "Medical - other" and "Surgical - other". General Practice was not included as this study was looking specifically at doctors working within a hospital environment.

Contacting consultants through the Royal Colleges of the various specialities in England was deemed the best way to access the whole workforce as best as possible. They were approached and asked to distribute this among all of their registered consultants. This was a total of 16 Royal Colleges. Of those contacted, only the Royal College of Radiologists, Royal College of Pathologists, and the Royal College of Emergency Medicine felt unable to forward this survey to their members. However, we still had responses from consultants within these specialities, so we can assume they were made aware of this survey by other means. A follow-up message was sent to the Royal Colleges that did not respond to the initial enquiries.

The results were collated on Google’s Forms application. The survey was sent out at the start of November 2025, and the results were collated at the start of December 2025. These were then analysed, cross-matching various results against each other.

Ethical approval was sought from the Research and Development department at University Hospitals Coventry & Warwickshire. Following review, the study was classified as a service evaluation involving anonymous survey data from healthcare professionals and was deemed not to require formal NHS Research Ethics Committee approval. Participation was voluntary, no identifiable personal data were collected, and informed consent was obtained electronically at the start of the survey.

## Results

**Figure 1:**
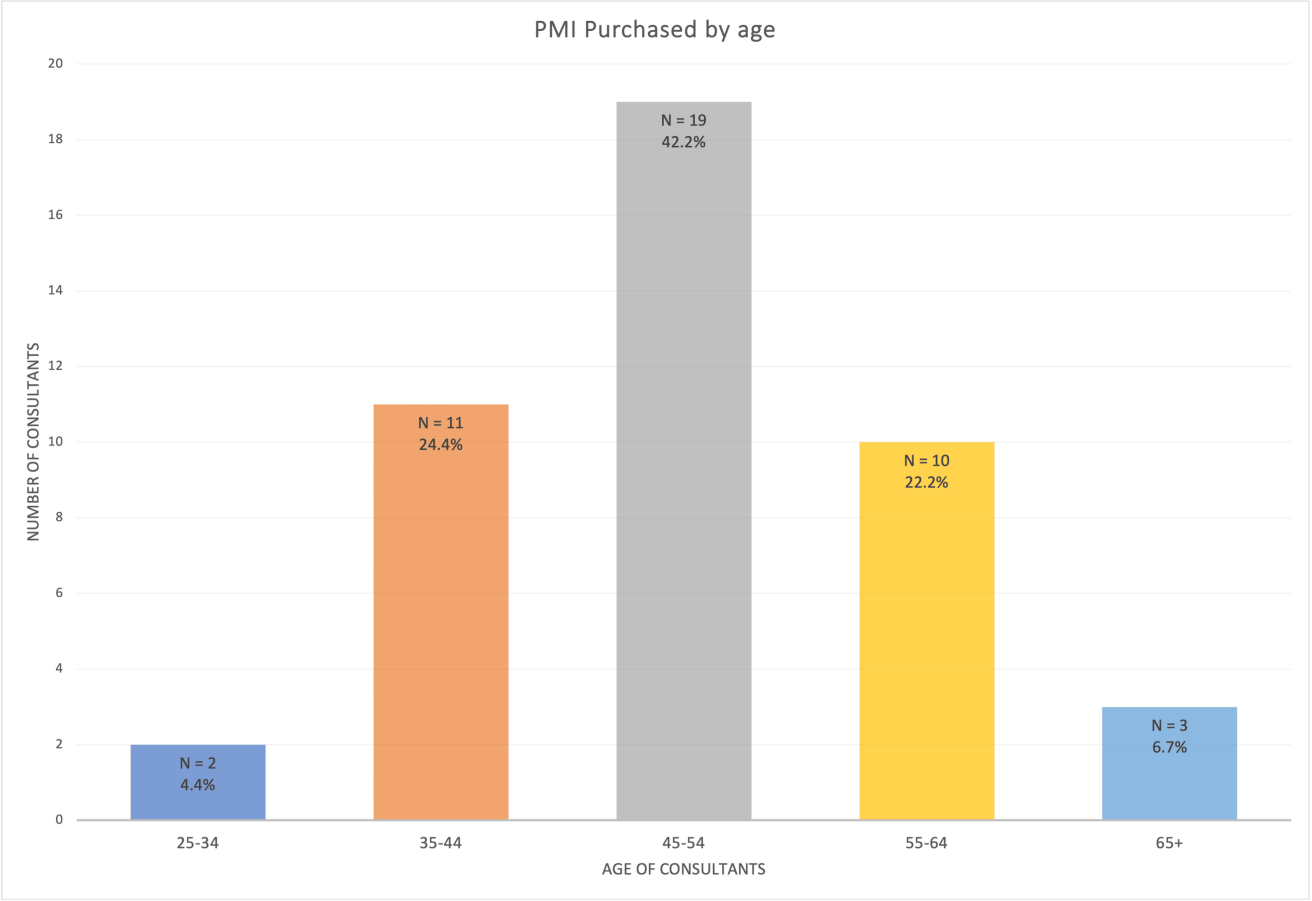
Rates of PMI purchase according to age PMI: private medical insurance

A total of 445 responses were received, with an estimated current cohort of 64,686 consultants in England [20]; this response rate is only 0.7%. A total of 45 (10.1%) reported having PMI. All respondents completed all sections of the survey. Among those, the most common age range was 45-54 years (19/45, 42.2%), with only 2 (4.4%) being 25-34 years old, and 3 (6.7%) being over 65 years old (Figure 1). A chi-square test showed that there was significant variation amongst these groups, χ²(4) = 23.19 (p < 0.001).

**Figure 2:**
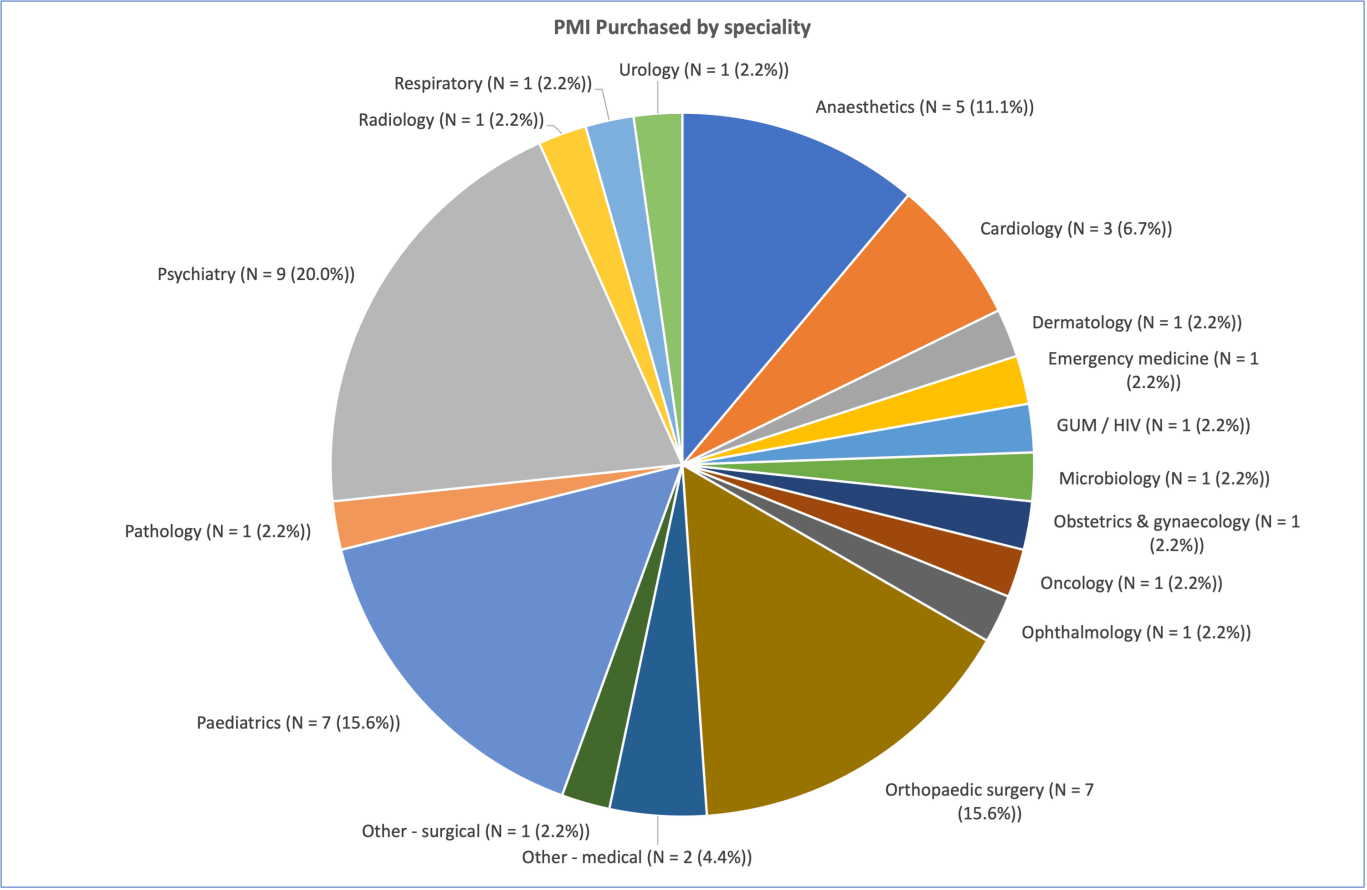
Total numbers of consultants who purchased PMI, by speciality (χ²(17) = 47.79, p < .001) PMI: private medical insurance; GUM: Genitourinary Medicine

Most of the consultants who had purchased PMI were psychiatrists (9/45, 20.0%), followed by orthopaedic and paediatric consultants (7/45, 15.6%) (Figure 2). The variance of this result was significant. A chi-square test was conducted on this data, χ²(17) = 47.79 (p < 0.001). In this study, it was found that psychiatrists, orthopaedic surgeons, and paediatric doctors had a significantly higher rate of PMI purchase than other specialities.

**Figure 3:**
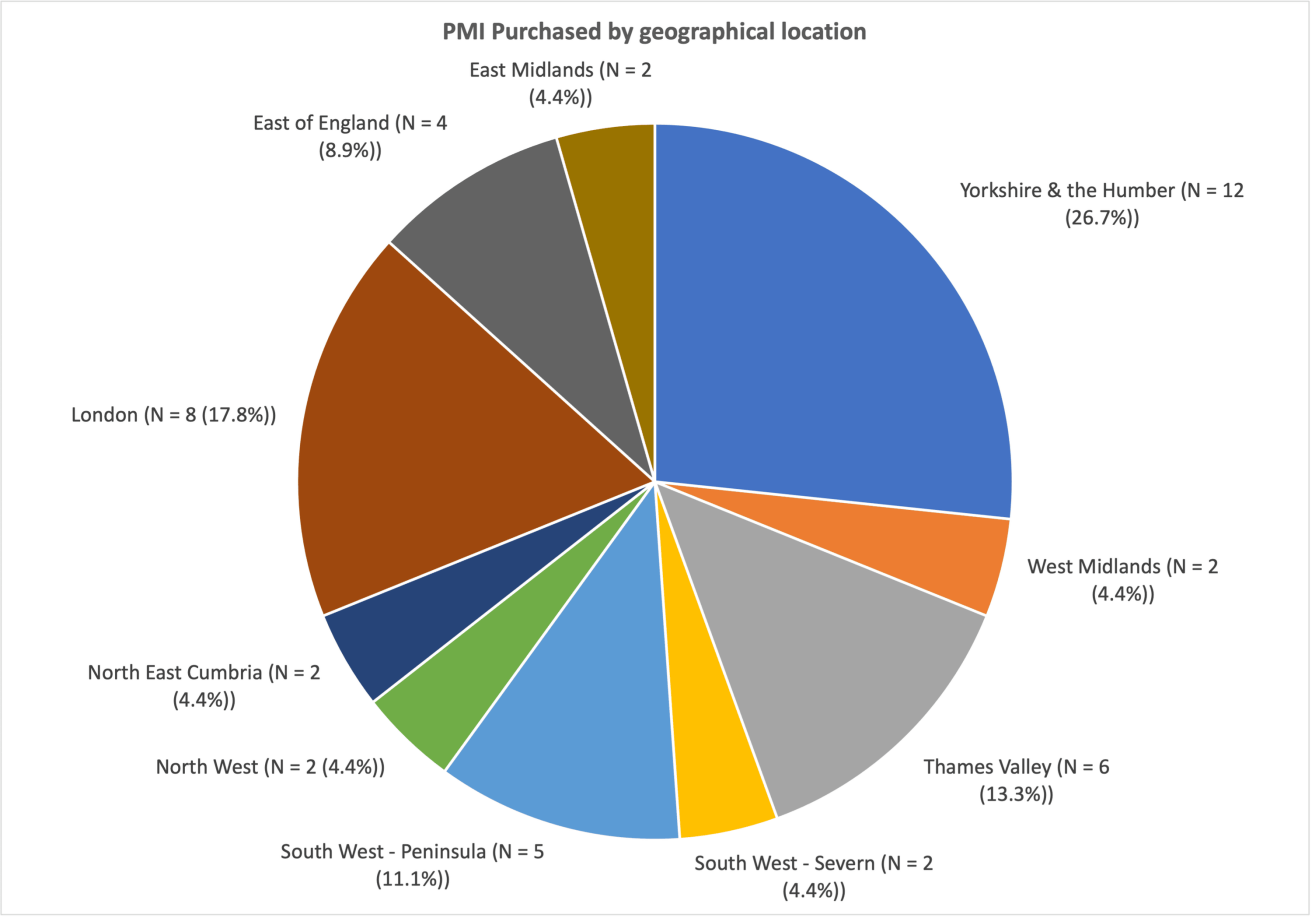
Total number of consultants who purchased PMI, by geographical location (χ²(9) = 23.85, p < 0.005) PMI: private medical insurance

The Yorkshire and Humber region had the greatest concentration of consultants purchasing PMI (12/45, 26.7%), followed by London (8/45, 17.8%) and South West - Peninsula (5/45, 11.1%) (Figure 3). A chi-square test showed a statistically significant association between region and PMI status, χ²(9) = 23.85 (p < 0.005), suggesting that the rates of PMI differed significantly across regions.

**Figure 4:**
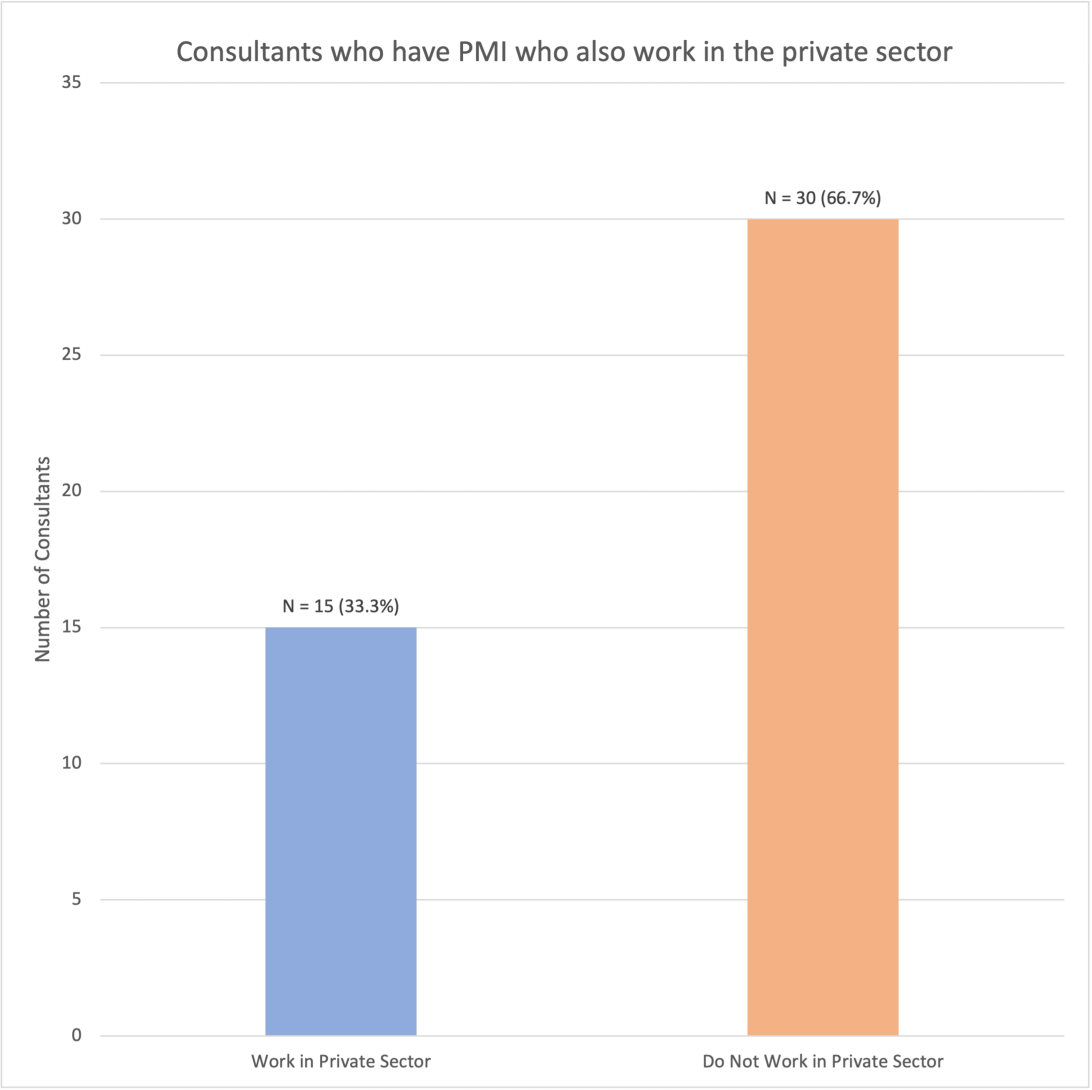
Rates of work within the private sector amongst consultants with PMI (χ²(1) = 6.45, p = 0.01) PMI: private medical insurance

Only 15 of the 45 (33.3%) consultants who reported having purchased PMI said that they worked in the private sector, with the remaining 30 saying they did not work privately (66.7%) (Figure 4). This was a significant difference (χ²(1) = 6.45, p = 0.01), suggesting that those who have purchased PMI are almost twice as likely to not work in the private sector themselves.

A total of 96 out of the total 445 (21.6%) consultants who replied said that they work in the private sector as well as the NHS. The majority were orthopaedic surgeons (14/96, 14.6%), followed by anaesthetists (11/96, 11.5%).

**Figure 5:**
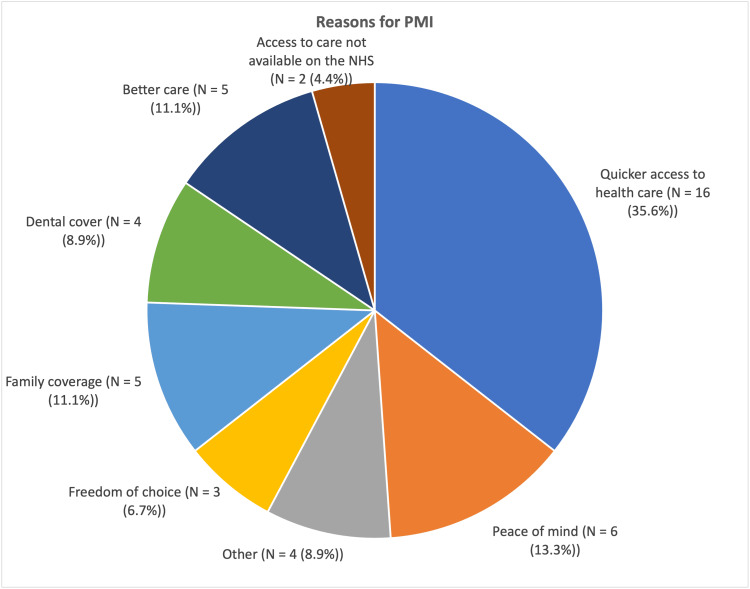
Reasons provided by consultants purchasing PMI, by total number of respondents PMI: private medical insurance

The most common reason reported by people for having PMI was quicker access to healthcare, with 16 of 45 people giving this reason (35.6%), significantly higher than any other reason (p < 0.0001). Peace of mind, family coverage, and better care were other frequent responses, totalling six, five, and five out of 45, respectively. Four people gave "Other" as the reason for purchasing their policy (Figure 5).

## Discussion

The NHS does not routinely provide PMI for its employees; therefore, the results should be compared to the proportion of the UK population who self-fund their insurance policies. However, it is estimated that only around 2.5% of the UK population has self-funded PMI [17].

This survey can only be taken in the context of an exploratory survey; however, it would suggest that NHS consultants have a higher rate of self-funded PMI than the general population. It is important to consider that the figures for the general population are not stratified by income, and therefore, the population data may be skewed when taking into account the population of people with less disposable income than NHS consultants. The reasons why this cohort of doctors may feel the need to invest in PMI will undoubtedly be broad; however, this study tried to delve into this to give a greater understanding of their perspectives and beliefs.

There appears to be a considerable variation in consultants who have PMI depending on the geographical area. It is not possible to ascertain if this is truly indicative and significant, due to the low numbers of the study. The relevance of this variation is not clear; however, this could be further analysed by including socioeconomic variation, as well as NHS target achievements by geographical area, and comparing these results to the results of our survey. With resources and provision of services varying amongst local integrated care boards, it is possible that this may be a factor in determining whether patients seek options above and beyond the care available in the NHS. However, this is purely speculative.

As RTTs have increased significantly in recent years [1], it is unsurprising that the most common reason for having PMI was for quicker access to healthcare. This is despite NHS consultants undoubtedly having an acute understanding of the NHS infrastructure and how best to navigate with utmost efficiency, a trait few in the general population would possess. It is possibly indicative of a widespread concern amongst the British population regarding access to healthcare. Other factors also appeared to be important to those who responded, including the slightly more vague "peace of mind". This could encompass many of the other factors; however, this may suggest a more overall uncertainty regarding NHS provision of care. Better care was given as another relatively common reason, which would suggest that those consultants believe the private sector offers better quality of care. This does not necessarily mean that the care in the NHS is inadequate. However, with the relative strains on NHS and private healthcare, it would not be unsurprising to find that overall quality of care for an individual may be better by some metrics in the private sector. This could be measured in numerous ways and is beyond the scope of this study.

With as little as 40% of English patients able to see an NHS dentist in the year to March 2024 [21], despite government incentives and efforts to improve access and provision since COVID-19, dental cover as the reason for PMI seems sensible to some as well. With 4 (8.9%) of those who have bought PMI citing dental cover as a reason for purchase, it is another area in which the UK appears to be falling short. An important consideration is that the assumed knowledge of system infrastructure does not apply in the context of dentistry and NHS hospital consultants, as the two are mostly quite distinct.

As a secondary outcome measure, we identified that around 21.6% of NHS consultants were working in the private sector. This was consistent with recent figures elicited from government documents during the recent COVID-19 preparedness inquiry [22]. This report also showed that approximately 90% of consultants who worked in the private sector also worked within the NHS.

The assumption that the rates of self-funded insurance in NHS consultants can be compared to those within the wider population may not be entirely reliable, as a proportion of the people with employer-funded insurance policies may have bought their own policy if it was not already provided for them through work. If this were true, it would increase the rate in the general population to an unknown figure.

Rising RTTs in the NHS are clearly an important factor for people considering PMI. This is evident not only regarding the wider public, but also healthcare professionals. This problem is multifactorial, with no simple solution; however, it has been publicly touted by governments over the years as a key area of concern that could be an area to improve. Several of these factors have been explored at the start of this study.

**Figure 6:**
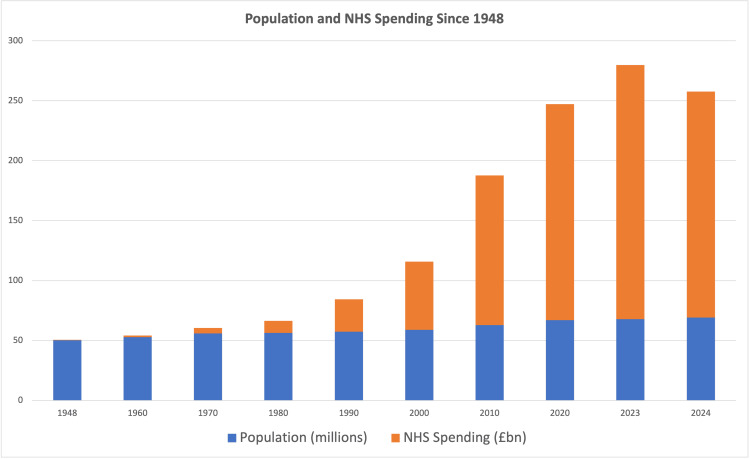
UK population and NHS spending increases since 1948

Despite funding and staffing increases significantly outstripping the rate of population growth (Figure 6), there is the perception that the NHS is not meeting its demands, in the media, in publications [23], and in the results of this study. However, it appears that the financial cost and staffing requirements of healthcare have potentially increased at a greater rate. It is important to note that the acquisition of PMI does not necessarily correlate with the holder’s personal opinion regarding support for an NHS system; at least in a Norwegian case study, the vast majority of the population believed that health services should mainly be a public responsibility [24].

Future work on this topic would need to include a greater proportion of the workforce, and could also include other healthcare professionals for a wider perspective. It would also be helpful to stratify the results against similar age and household income variables in both the NHS staff cohorts and the wider public. However, the data on the wider population household income is not available at present, to our knowledge.

There are several limitations present in this study, and with this being predominantly an exploratory survey, the results must be taken with caution. Most significantly, the response rate means that the outcomes are indicative of perceptions of NHS consultants, but are in no way conclusive evidence. This survey indicates the position of just 0.7% of this cohort, and must be considered only as a possible reflection of the group as a whole. There is little further we could do to encourage participation in this study, and it is entirely voluntary. However, further engagement with the various Royal Colleges in the future may allow us to elicit more accurate information. It is possible that there is an element of response bias, and this may skew our results; furthermore, the statistical analysis is based on relatively small numbers and must also be considered cautiously as well. Both of these would benefit from engagement from a larger proportion of the workforce. We also cannot confirm that all Royal Colleges sent out the survey at equal time points, if at all. This would need to be clarified to minimise bias in future surveys.

## Conclusions

To conclude, NHS consultants may be more likely to have PMI than the general population. The most common reason for this is faster access to healthcare services. This suggests that in the current climate, a proportion of our most senior NHS medical staff may have profound concerns about timely access to care, to such an extent that they have purchased PMI in order to compensate for this.
